# Stabilizing Role of Platelet P2Y_12_ Receptors in Shear-Dependent Thrombus Formation on Ruptured Plaques

**DOI:** 10.1371/journal.pone.0010130

**Published:** 2010-04-12

**Authors:** Reyhan Nergiz-Unal, Judith M. E. M. Cosemans, Marion A. H. Feijge, Paola E. J. van der Meijden, Robert F. Storey, J. J. J. van Giezen, Mirjam G. A. oude Egbrink, Johan W. M. Heemskerk, Marijke J. E. Kuijpers

**Affiliations:** 1 Department of Biochemistry, Cardiovascular Research Institute Maastricht (CARIM), Maastricht University, Maastricht, The Netherlands; 2 Department of Cardiovascular Science, University of Sheffield, Sheffield, United Kingdom; 3 AstraZeneca R&D Mölndal, Mölndal, Sweden; 4 Department of Physiology, Cardiovascular Research Institute Maastricht (CARIM), Maastricht University, Maastricht, The Netherlands; Hong Kong University, Hong Kong

## Abstract

**Background:**

In most models of experimental thrombosis, healthy blood vessels are damaged. This results in the formation of a platelet thrombus that is stabilized by ADP signaling via P2Y_12_ receptors. However, such models do not predict involvement of P2Y_12_ in the clinically relevant situation of thrombosis upon rupture of atherosclerotic plaques. We investigated the role of P2Y_12_ in thrombus formation on (collagen-containing) atherosclerotic plaques *in vitro* and *in vivo*, by using a novel mouse model of atherothrombosis.

**Methodology:**

Plaques in the carotid arteries from *Apoe* ^−/−^ mice were acutely ruptured by ultrasound treatment, and the thrombotic process was monitored via intravital fluorescence microscopy. Thrombus formation *in vitro* was assessed in mouse and human blood perfused over collagen or plaque material under variable conditions of shear rate and coagulation. Effects of two reversible P2Y_12_ blockers, ticagrelor (AZD6140) and cangrelor (AR-C69931MX), were investigated.

**Principal Findings:**

Acute plaque rupture by ultrasound treatment provoked rapid formation of non-occlusive thrombi, which were smaller in size and unstable in the presence of P2Y_12_ blockers. *In vitro*, when mouse or human blood was perfused over collagen or atherosclerotic plaque material, blockage or deficiency of P2Y_12_ reduced the thrombi and increased embolization events. These P2Y_12_ effects were present at shear rates >500 s^−1^, and they persisted in the presence of coagulation. P2Y_12_-dependent thrombus stabilization was accompanied by increased fibrin(ogen) binding.

**Conclusions/Significance:**

Platelet P2Y_12_ receptors play a crucial role in the stabilization of thrombi formed on atherosclerotic plaques. This P2Y_12_ function is restricted to high shear flow conditions, and is preserved in the presence of coagulation.

## Introduction

Rupture of an atherosclerotic plaque and occlusive arterial thrombus formation is the major cause of acute cardiovascular incidents and deaths in the Western countries [1]. The development of a thrombus is a complex and dynamic process, involving platelet aggregation, generation of thrombin and formation of a network of fibrin, to strengthen the platelet aggregate and stabilize the thrombus [2], [3]. *In vivo* mouse models of experimental thrombosis have indicated a major role of platelet receptors in the process of thrombus formation and stabilization [4]. However, the studies have been carried out by damaging healthy mouse arteries in an artificial way, *e.g.* by laser-induced tissue ablation, free radical-generating agents or mechanical disruption. Thrombus formation in diseased, atherosclerotic arteries following plaque rupture has hardly been investigated.

Ruptured atherosclerotic plaques expose several platelet-adhesive and -activating components, such as collagen types I and III, von Willebrand factor (VWF), lysophosphatidic acid, thrombospondin, fibronectin, vitronectin, fibrin/fibrinogen and oxidized low density lipoprotein [5], [6], [7], [8], [9], [10]. In addition, plaques contain tissue factor which, upon de-encryption, activates the extrinsic coagulation system [5], while the intrinsic system of factor XII activation is triggered via collagen [11]. To which extent each of these plaque components contribute to thrombus formation is unclear, though the platelet-activating roles of VWF and collagen are well-described [1]. Collagen-bound VWF mediates the initial tethering and transient adhesion of platelets via glycoprotein (GP)Ib-V-IX. Stable platelet adhesion to VWF is achieved via integrin α_IIb_β_3_ and adhesion to collagen via the platelet receptors, GPVI and integrin α_2_β_1_ [2], [12], [13]. Recent *in vitro* studies suggest that these receptors also mediate platelet adhesion to collagen in damaged plaques [5], [6]. Subsequent activation responses of adhered platelets include mobilization of cytosolic Ca^2+^, secretion of autocoids like ADP, activation of integrin α_IIb_β_3_ (fibrinogen receptor) and formation of pseudopods, all of which events help to recruit circulating platelets into a thrombus [2], [14]. Part of the activated platelets become procoagulant by Ca^2+^-dependent exposure of phosphatidylserine on their surface, which is required for local thrombin generation [15].

The autocrine agent ADP activates platelets via two receptors, P2Y_1_ and P2Y_12_; the first of which mediates shape change and initiates platelet aggregation, while the latter is required for complete aggregation [16]. The importance of P2Y_12_ was revealed by the observation that the damage of arteries in P2Y_12_-deficient mice resulted in markedly delayed and unstable thrombus formation [17]. Mechanistically, we and others have shown that continuous signaling via P2Y_12_ is required to prevent platelet disaggregation and to maintain α_IIb_β_3_ in its active conformation [18], [19]. The P2Y_12_ receptor also has a role in platelet procoagulant activity by potentiating tissue factor-induced thrombin generation via sustained Ca^2+^ mobilization [20], [21].

In the present paper, we used a recently developed mouse model of thrombus formation on acutely ruptured plaques to study the role of P2Y_12_ receptors in atherothrombosis. We investigated effects of the reversible P2Y_12_ antagonists, ticagrelor and cangrelor, not only using this *in vivo* model, but also in perfusion studies of whole (P2Y_12_-deficient) mouse or human blood perfused over collagen-containing plaque material. The results point to a P2Y_12_-dependent stabilization of thrombus formation at high shear, that is maintained under conditions of coagulation. This observation may have important implications for antithrombotic treatment in patients.

## Materials and Methods

### Materials

Fibrillar Horm collagen (type I) was purchased from Nycomed Pharma (Munich, Germany). Native fibrillar type I collagen was prepared from bovine tendon with minimal protease treatment, as described [22]. Fibrinogen labeled with Oregon Green (OG) 488 was from Invitrogen (Carlsbad, CA, USA). Carboxyfluorescein diacetate succinimidyl ester (CFSE) was from Molecular Probes (Leiden, the Netherlands). Rat-anti-mouse CD62 labeled with FITC (Wug.E9) and rat-anti-mouse GPIIbIIIa (JON/A) labeled with PE were from Emfret Analytics (Würzburg, Germany). Cangrelor (AR-C69931MX) and ticagrelor (AZD6140) were kindly provided by AstraZeneca (Mölndahl, Sweden). Ketamine and xylazine were from Eurovet (Bladel, the Netherlands). Sources of other materials are described elsewhere [23].

### Animals

Wild type C57BL/6 mice (12 weeks old) and *Apoe* ^−/−^ mice on C57BL/6 background (4 weeks old) were obtained from Charles River (Maastricht, The Netherlands). The *Apoe* ^−/−^ mice were fed a Western-type diet with 0.15% cholesterol for 18–20 weeks, *i.e*. until plaques had developed in the carotid arteries near the bifurcation. Mice homozygously deficient in P2Y_12_ receptors [24] and corresponding wild types (C57BL/6 background) were bred and housed, as described previously [25]. Animal experiments were approved by the research ethics committees of the universities of Maastricht (The Netherlands) and Sheffield (UK).

### *In vivo* plaque rupture and measurement of thrombus formation

Acute rupture of plaques in the carotid arteries was provoked by targeted ultrasound treatment, as described before [26]. Briefly, *Apoe* ^−/−^ mice fed with cholesterol-containing diet were anesthetized by subcutaneous injection of ketamine and xylazine (0.1 mg/g and 0.02 mg/g body weight, respectively). The carotid arteries were carefully dissected free from surrounding tissue, and the anesthetized animals were injected intravenously with 10% CFSE-labeled platelets, which were obtained from a donor mouse of the same genotype. The mice were then injected with vehicle solution, ticagrelor or cangrelor, as described below. Body temperature was held at 37°C during all procedures.

Using brightfield illumination and intravital microscopy, a plaque was selected in one of the carotid arteries near the bifurcation. The tip (0.5 mm diameter) of a titanium ultrasound probe was then placed at the plaque shoulder region. Rupture of the plaque was induced by ultrasound application during 10 s at 6 kHz, using a VibraCell VCX130 processor (Sonics, Newtown, CT, USA). Thrombus formation was recorded in real time by capturing 12-bit fluorescence images at 33 Hz during at least 10 min, using a back-thinned electron multiplier C9100-12 EM-CCD camera (Hamamatsu, Japan) at fixed gain settings. Local rupture of the plaques was verified by two-photon laser scanning microscopy and histological staining of sections of the carotid arteries [26].

Time-series of fluorescence images were analyzed using Wasabi (Hamamatsu) software. Per region of interest (ROI), corresponding to the site of thrombus formation, total pixel intensity was calculated and corrected for background intensity. To quantify thrombus size, digital images taken at specific time points were processed using ImagePro software (Media Cybernetics, Silverspring, MD, USA). Within the carotid artery, two similar ROIs were defined, one representing the thrombus area and an adjacent ROI representing the background. A threshold level was set by eliminating all pixels with intensity lower than 99.0% of the pixels of the background ROI. Intensities (gray levels) of all pixels in the thrombus ROI were then integrated. No image processing was applied.

### *In vivo* administration of P2Y_12_ antagonist and blood collection

Anesthetized mice were injected intravenously with vehicle solution (5% mannitol in saline), ticagrelor (AZD6140) or cangrelor (AR-C69931MX). Ticagrelor was infused at a dose of 420 µg/kg for 1 min, followed by 60 µg/kg/min for 15 min; cangrelor was infused at 3 µg/kg/min for 15 min, and infusions were continued during the experiment. Mice for control experiments were bled retro-orbitally under anesthetics after 15 min of infusion of vehicle solution, ticagrelor or cangrelor. Mouse blood was collected into 40 µM PPACK and 5 U/ml heparin or trisodium citrate, as indicated [13]. Platelet-rich plasma (PRP) was isolated and used for platelet activation analysis. Human blood taken by venipuncture, was collected into 40 µM PPACK or 12.9 mM trisodium citrate, as indicated.

Flow-cytometric evaluation of platelets with active α_IIb_β_3_ or P-selectin exposure was performed by activation with ADP (40 µM) or convulxin (100 ng/ml) in 20x diluted whole mouse blood, followed by labeling with JON/A mAb or anti-CD62 mAb, respectively. Detection of fluorescence was with a FACScan flow cytometer, equipped with an argon laser (Becton-Dickinson, Franklin Lakes, NJ). The percentage of positive cells was analyzed using WinMDI 2.8 software (http://facs.scripps.edu).

### Whole blood perfusion experiments

Human and mouse blood samples were perfused through a parallel-plate transparent flow chamber containing a coverslip, coated with either plaque material or collagen, as described [5]. PPACK-anticoagulated blood was used for perfusion experiments without coagulation, respectively; ADP (10 µM) was co-perfused, as indicated [18]. Coagulation was introduced by co-perfusion of human or mouse citrate-anticoagulated blood with 0.1 volume of CaCl_2_ (75 mM), MgCl_2_ (37.5 mM) and tissue factor (20 pM) in Hepes buffer pH 7.45 (NaCl 110 mM, glucose 10 mM, Hepes 5 mM, KCl 2.7 mM and 0.1% bovine serum albumin). Wall shear rate was 300–1000 s^−1^, as indicated. After blood perfusion, thrombi on coverslips were rinsed with Hepes buffer supplemented with 2 mM CaCl_2_, 2 mM MgCl_2_ and heparin (1 U/ml). Recording of real-time videos and capturing of phase-contrast images was with a non-confocal microscopic system [13]. Videos were analyzed offline for embolizing events (single or clustered platelets) per aggregate [18]. Randomly captured images were analyzed for surface area coverage, area distribution of individual segmented features or integrated fluorescence intensity with Image-Pro (ImagePro, Silver Spring MD, USA) or Metamorph software (MDS Analytical Technologies, Downingtown, PA, USA) [27]. In specific experiments, OG488-fibrinogen (25 µg/ml) was added to human or (P2Y_12_-deficient) mouse blood and, after perfusion over collagen or plaque material, fluorescence accumulation was detected by confocal laser scanning microscopy. Confocal images were recorded off-line with a BioRad 2100 multiphoton system at fixed settings of argon laser power, scanning rate and photomultiplier gain [15].

### Coating of plaque material

Human atherosclerotic plaques were collected at autopsy from a carotid artery and used in compliance with institutional guidelines (Department of Pathology, Maastricht University). Permission was obtained from the local Medical Ethics Committee. Murine atherosclerotic plaques were collected from the aortic arches of 6 *Apoe* ^−/−^ mice, fed with a Western-type diet with 0.15% cholesterol for 18–20 weeks. After resection, atherosclerotic specimens were frozen into liquid nitrogen and stored at −80°C. Thawed human and mouse atherosclerotic tissues were homogenized in phosphate-buffered saline. Plaque homogenates were pooled at 165 mg wet tissue weight/ml, as described [5]. Coverslips were coated with plaque material at a density of 170 µg wet weight/cm^2^.

### Cone and plate(let) experiments

An Impact-R cone and plate(let) analyzer (CPA, DiaMed, Cressier, Switzerland) was used to evaluate platelet adhesion and aggregation on a surface under defined flow conditions [28]. Citrate-anticoagulated blood (130 µl) was placed in a polystyrene well and subjected to a shear of 500–5000 s^−1^ for 2 min. The platelet aggregates were washed and stained by May-Grünwald stain, according to the manufacturer's instructions (DiaMed). Platelet deposition was evaluated by an image analyzer system connected to the microscope, measuring the average size of the aggregates in µm^2^.

### Thrombin generation experiments

Thrombin generation was measured in citrate-anticoagulated human or mouse PRP (1×10^8^ platelets/ml), as before [29]. Briefly, PRP was preincubated with inhibitors, and then treated with ADP (20 µM) or vehicle for 10 min. Samples (4 volumes) were pipetted into a polystyrene 96-wells plate (Immulon 2HB, Dynex Technologies, Chantilly, VA, USA), already containing 1 volume of buffer A (20 mM Hepes, 140 mM NaCl and 0.5% bovine serum albumin) and tissue factor (6 pM). Coagulation was started by adding 1 volume of buffer B (2.5 mM Z-GGR-AMC, 20 mM Hepes, 140 mM NaCl, 100 mM CaCl_2_ and 6% bovine serum albumin). Samples were run at least in duplicate. First-derivative curves of accumulation of fluorescence in human plasma were converted into nM thrombin using a human calibrator.

### Statistical analysis

Data are presented as means ± SE. Groups were compared using the non-parametric Mann-Whitney U test (one-tailed) using the statistical package for social sciences (SPSS 15.0, Chicago, IL, USA). Size distribution of platelet aggregates was evaluated by χ^2^ analysis [27]. A p-value below 0.05 was considered significant.

## Results

### Inhibition of mouse P2Y_12_ receptors causes unstable thrombus formation after rupture of an atherosclerotic plaque

**Figure 1:**
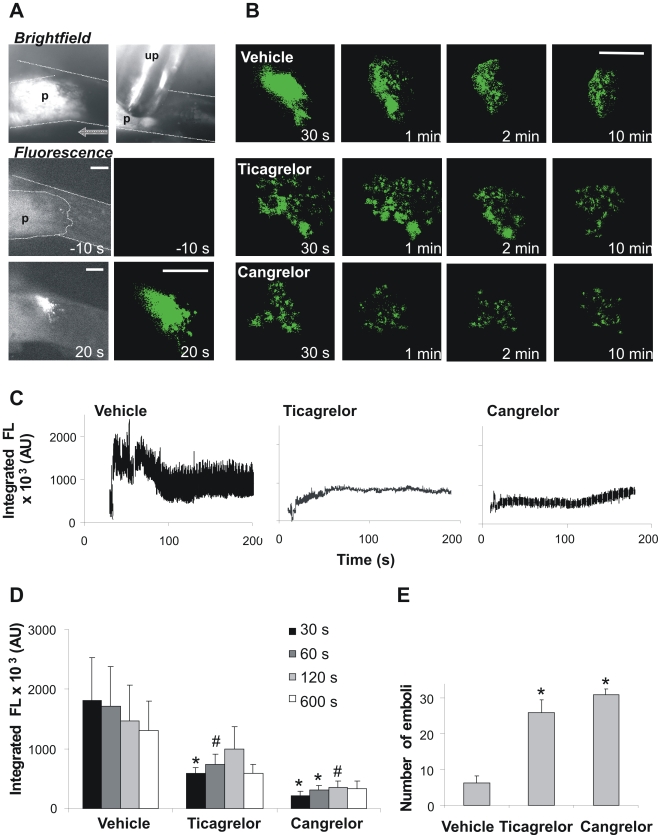
Inhibition of P2Y_12_ receptors causes unstable thrombus formation on acutely ruptured plaques in mice. *Apoe* ^−/−^ mice were infused with vehicle solution as a control. Other mice were infused with ticagrelor (210 µg/kg for 1 min, followed by 30 µg/kg/min during the experiment). In the third group of mice, cangrelor was continuously infused at 3 µg/kg/min. Infusion of ticagrelor and cangrelor started at 15 min before ultrasound treatment. (A) Brightfield image of a carotid plaque (p) with ultrasound probe (up). Further, raw fluorescence images (left) and threshold masked images (right) of CFSE-labeled platelets before and after ultrasound treatment. Dotted area indicates location of carotid artery (bars, 100 µm). Time stamps point to images at baseline (−10 s) or after plaque rupture (20 s). (B) Representative threshold masked images of thrombi on ruptured plaques of mice infused with vehicle, ticagrelor or cangrelor (bar, 100 µm). Note the microthrombi with P2Y_12_ antagonist formed within 1 min of ultrasound treatment. (C) Time-courses of integrated CFSE fluorescence intensity above background (arbitrary units, AU) of representative thrombi formed. (D) Quantification of thrombus size at various time points after ultrasound treatment. Data are integrated fluorescence intensities from threshold masked images. (E) Number of fluorescent emboli shed during 3 min after plaque rupture. Data are means ± SE (*n* = 3–8), *p<0.05 and ^#^ *p*<0.1 compared to vehicle.

To study the role of platelet P2Y_12_ in atherothrombosis, we used a recently established mouse model of acute plaque rupture [26]. Mice deficient in ApoE were fed a cholesterol-enriched diet for 18–20 weeks. Plaque-containing carotid arteries were dissected free from surrounding tissue, and CFSE-labeled *Apoe* ^−/−^ platelets were injected to enable measurement of fluorescence by intravital microscopy. In control animals infused with vehicle solution, targeted treatment with an ultrasound probe resulted in acute rupture and subsequent non-occlusive thrombus formation (Fig. 1A). As described, this thrombotic process relies on GPVI-induced platelet activation as well as on thrombin generation and coagulation [26]. Mice were infused with effective concentrations of one of the reversible P2Y_12_ antagonists, ticagrelor or cangrelor. In blood samples taken from some of these mice, it was checked that the ticagrelor or cangrelor interventions abolished ADP- and collagen-induced platelet aggregation (data not shown). In other mice, the infusion of either P2Y_12_ antagonist greatly suppressed platelet accumulation induced by carotid plaque rupture, in that only loose thrombi appeared, which consisted of single-layered fluorescent platelets, in contrast to the compact bright fluorescent thrombi in vehicle-treated mice (Fig. 1B). The reduced thrombus formation was also apparent from time-courses of integrated fluorescence intensity after plaque rupture (Fig. 1C). Similarly as described [26], in vehicle-treated mice, plaque rupture caused a biphasic pattern of rapid increase and slower decline in fluorescence accumulation. Only few single platelets were seen to leave the thrombus in the declining phase. Since fluorescence bleaching was relatively low, the declining phase had a different cause, likely contraction of the platelet-fibrin thrombus.

Off-line quantification of images showed that P2Y_12_ inhibition with either antagonist reduced the size of thrombi compared to the vehicle control (Fig. 1D). Video analysis further revealed that the thrombi formed in the presence of P2Y_12_ inhibitor were relatively unstable. With ticagrelor or cangrelor present, 4–5 fold more emboli were shed from thrombi in comparison to control mice (Fig. 1E). Together, these data pointed to a marked inhibiting effect of the reversible P2Y_12_ antagonists on *in vivo* thrombus formation following plaque rupture.

### Absence of functional P2Y_12_ receptors suppresses thrombus formation and thrombin generation, but increases disaggregation

**Figure 2:**
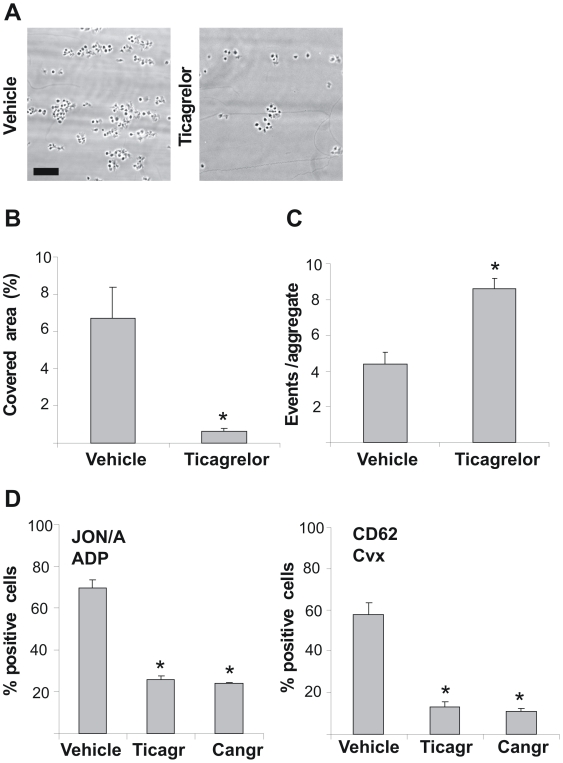
Inhibition of murine P2Y_12_ receptors diminishes thrombus formation and provokes platelet disaggregation under flow. *Apoe* ^−/−^ mice were infused with vehicle solution, ticagrelor or cangrelor (see Figure 1). (A–C) PPACK/heparin anticoagulated blood from treated mice was perfused over native type I collagen at a shear rate of 1000 s^−1^ for 4 min. (A) Representative phase-contrast images after 4 min perfusion (bar = 20 µm). (B) Quantitative effect of ticagrelor on surface area coverage by platelets. (C) Average number of disaggregation events measured from a preformed aggregate during flow. (D) Flow-cytometric evaluation in whole blood of platelets with active α_IIb_β_3_ (JON/A mAb) by activation with ADP (20 µM), and of platelets exposing P-selectin (anti-CD62 mAb) by activation with convulxin (Cvx, 10 ng/ml). Bars give percentages of positive platelets. Data are means ± SE (*n* = 3–4), *p<0.05.

To study the mechanism by which P2Y_12_ contributes to thrombus stabilization, we performed flow chamber experiments over native type I collagen with blood from *Apoe* ^−/−^ mice which were infused with ticagrelor. Perfusion of blood from ticagrelor-treated mice resulted in diminished thrombus formation on collagen (Fig. 2A). The treatment significantly reduced platelet deposition, while it increased the disaggregation events (Fig. 2B, C). Flow cytometry using whole blood from mice infused with ticagrelor demonstrated near complete inhibition of α_IIb_β_3_ activation in response to ADP and of P-selectin expression in response to low convulxin (Fig. 2D).

**Figure 3:**
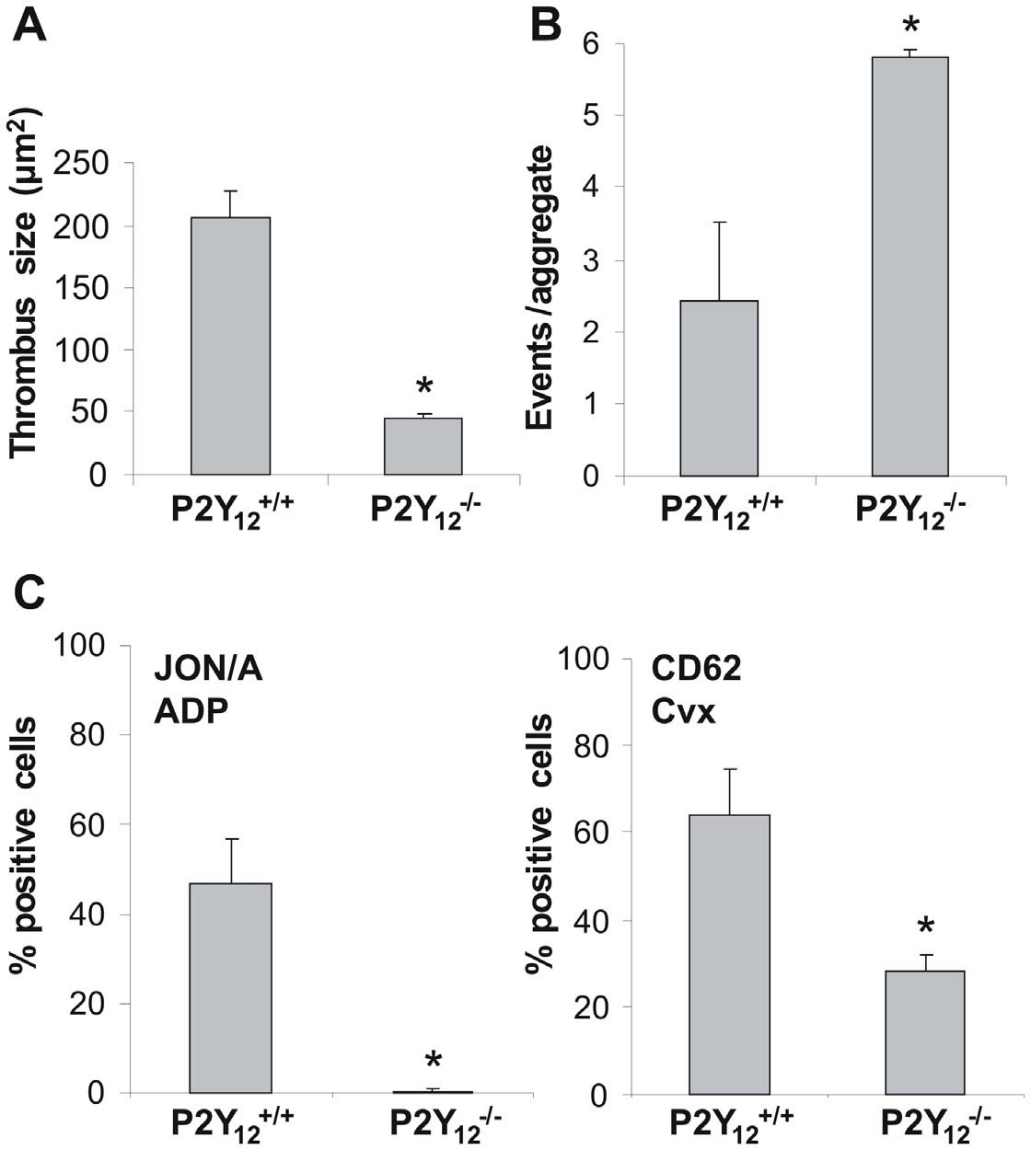
Deficiency in mouse P2Y_12_ receptors diminishes thrombus formation on collagen and provokes disaggregation under flow. Blood from P2Y_12_ deficient (P2Y_12_^−/−^) or wild type (P2Y_12_^+/+^) mice was perfused over native type I collagen at 1000 s^−1^ for 4 min in the presence of 10 µM ADP. (A) Quantitative effect of P2Y_12_ deficiency on surface area coverage by platelets. (B) Average number of disaggregation events measured from a preformed aggregate during flow (*n* = 3). (C) Flow-cytometric evaluation of ADP-stimulated platelets with active α_IIb_β_3_ (JON/A mAb) and of convulxin-stimulated platelets exposing P-selectin (anti-CD62 mAb) in whole blood. Experiments were performed as in Fig. 2. Data are means ± SE (*n* = 5–6), *p<0.05.

To confirm the role of P2Y_12_ in these platelet responses, experiments were performed with blood from P2Y_12_-deficient mice. Perfusion of blood from these mice also resulted in diminished thrombus formation (Fig. 3A), accompanied by increased disaggregation events (Fig. 3B). Furthermore, flow cytometry using whole blood from P2Y_12_-deficient mice demonstrated complete inhibition of α_IIb_β_3_ activation in response to ADP and of P-selectin expression in response to low convulxin (Fig. 3C). Ticagrelor was without further effect on these responses of P2Y_12_-deficient platelets (not shown).

**Figure 4:**
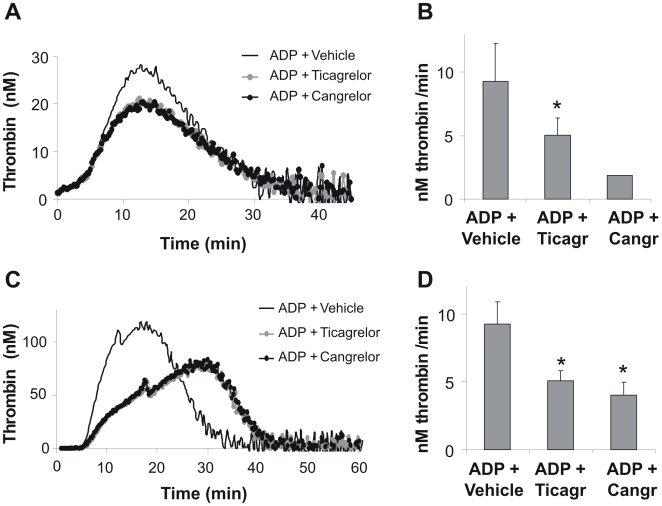
Procoagulant role of P2Y_12_ receptors in platelet-dependent thrombin generation. Mouse (A, B) or human (C, D) PRP was preincubated with vehicle, ticagrelor (20 µM) or cangrelor (20 µM), and stimulated with ADP (20 µM). Thrombin generation was measured after triggering with tissue factor (1 pM) and CaCl_2_ (16.7 mM). (A, C) Representative thrombin generation curves of PRP stimulated with ADP alone, or ADP in combination with P2Y_12_ inhibitor. (B, D) Quantitative effect of ticagrelor or cangrelor on ADP-stimulated rate of thrombin formation. Data are means ± SE (*n* = 3–6), *p<0.05 compared to ADP + vehicle.

Since enhancement of platelet-dependent thrombin generation is an established outcome of P2Y_12_ signaling [20], effects of P2Y_12_ antagonism on thrombin generation were investigated in mouse and human PRP. Dose-response experiments demonstrated that 20 µM ticagrelor or cangrelor were maximally effective (data not shown). At this concentration, either antagonist partly reduced the stimulating effect of ADP on thrombin peak levels in mouse and human PRP (Fig. 4A, C). Since in particular the rate of thrombin generation is an important parameter in flow-dependent thrombus formation, we quantified the slope of the thrombin generation curves for effects of P2Y_12_ inhibition. both ticagrelor and cangrelor markedly suppressed this slope in mouse and human PRP (Fig. 4B, D). We reasoned that this contribution of P2Y_12_ may play a role in thrombus stabilization by increasing the availability of thrombin to activate platelets and form fibrin fibers.

### Inhibition of P2Y_12_ impairs thrombus formation and stabilization at high but not low shear rate

**Figure 5:**
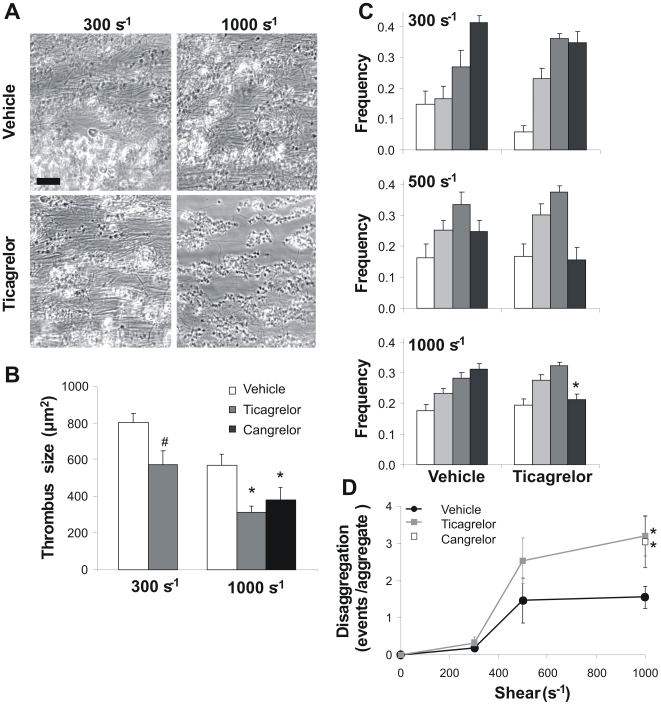
Inhibition of P2Y_12_ receptors affects collagen-dependent thrombus formation at high but not low shear rate. Citrate-anticoagulated human blood, recalcified with CaCl_2_/MgCl_2_ in the presence of 2 pM tissue factor, was perfused over Horm type I collagen at a shear rate of 300 or 1000 s^−1^. Blood samples were preincubated with vehicle, ticagrelor (20 µM) or cangrelor (10 µM), as indicated. (A) Representative phase-contrast images after 4 min of perfusion (bar = 20 µm). (B) Average size of thrombi formed in treated blood samples at different shear rates. (C) Frequency distribution of feature size on coverslips; estimated numbers of platelets per feature (aggregate) were: 9–24 (white), 24–75 (light gray), 75–400 (dark gray) and >400 (black). (D) Number of disaggregation events from individual aggregates in the 4th minute of blood perfusion at 300 or1000 s^−1^. Data are means ± SE (*n* = 3–9), *p<0.05 compared to vehicle.

The clinical efficacy of P2Y_12_ antagonists may depend on the local shear rate, which can vary considerably at arterial sites of vulnerable atherosclerotic plaques. To investigate this, human blood was perfused at low and high shear rate over a collagen-containing surface under physiological conditions allowing coagulation. Under control conditions in the absence of P2Y_12_ inhibitors, stable thrombi containing platelets and fibrin were formed at shear rates of 300 to 1000 s^−1^ (Fig. 5A). Fibrin fibers were most clearly visible between adjacent thrombi. In the presence of ticagrelor or cangrelor, much smaller thrombi were formed. Especially at 1000 s^−1^, fibrin fiber formation and the average thrombus size were drastically reduced with ticagrelor (Fig. 5B). Analysis of the size distribution patterns on coverslip showed that ticagrelor suppressed the formation of larger thrombi (>400 platelets) only at this higher shear rate (Fig. 5C). Interestingly, this was accompanied by a twofold increase in disaggregation events, again only at 1000 s^−1^ (Fig. 5D). Similar effects of P2Y_12_ inhibition on thrombus stabilization at high shear were seen in flow studies without coagulation (not shown). Together, these experiments indicated that, under conditions of high shear and coagulation, P2Y_12_ antagonism suppressed the formation of stable, fibrin-containing thrombi.

**Figure 6:**
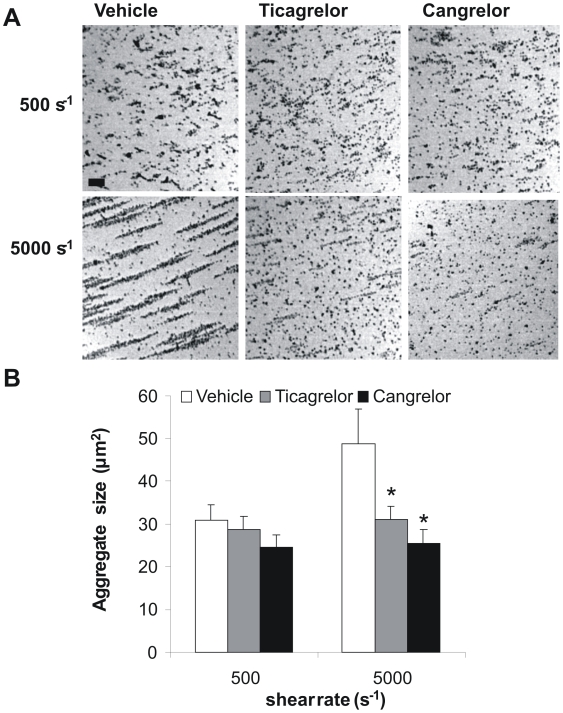
Inhibition of P2Y_12_ receptors reduces aggregate size at high but not low shear in cone and plate(let) analyzer. Citrate-anticoagulated human blood was preincubated with vehicle, ticagrelor (20 µM) or cangrelor (10 µM) for 10 min. Blood samples were subjected to a shear rate of 500 or 5000 s^−1^ for 2 min in a cone and plate(let) analyzer (CPA). (A) Representative images of platelet aggregates formed on the surface after 2 min (bar = 20 µm). (B) Average aggregate size in µm^2^. Data are means ± SE (*n* = 4–6), *p<0.05 compared to vehicle.

To confirm the shear-dependent effect of P2Y_12_ inhibition, additional whole blood experiments were performed with a cone and plate(let) analyzer. This test evaluates platelet adhesion and aggregation to a surface under defined shear conditions [28]. At a low shear rate of 500 s^−1^, the size of the platelet aggregates was not influenced by ticagrelor or cangrelor (Fig. 6A, B). However, at 5000 s^−1^, the larger size aggregates became greatly sensitive to P2Y_12_ inhibition. This experiment hence supports the conclusion that P2Y_12_ contributes to thrombus formation especially at high shear rate.

### Inhibition or deficiency of P2Y_12_ suppresses thrombus formation on plaques under high shear flow

**Figure 7:**
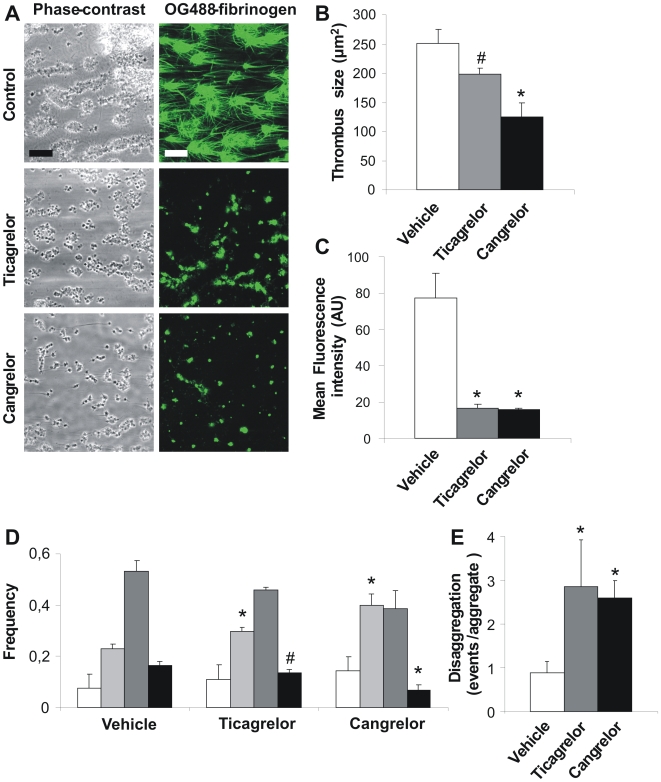
Inhibition of P2Y_12_ receptors affects plaque-induced thrombus formation at high shear rate. Citrate-anticoagulated human blood was supplemented with OG488-fibrinogen (25 µg/ml), recalcified with CaCl_2_/MgCl_2_ in the presence of 2 pM tissue factor and perfused over homogenized human plaque material at 1000 s^−1^ for 8 min. Blood was pre-incubated with ticagrelor (20 µM) or cangrelor (10 µM) as indicated. (A) Representative phase-contrast and fluorescence images (bar = 20 µm). (B) Average size of thrombi after perfusion. (C) Mean fluorescence intensity from fibrin(ogen)-binding platelets and thrombi. (D) Histograms of features on surface; the estimated numbers of platelets per feature were 9–24 (white), 24–75 (light gray), 75–400 (dark gray) and >400 (black). (E) Disaggregation events measured per platelet aggregate per min. Data are means ± SE (*n* = 3), *p<0.05, ^#^ *p* = 0.06 compared to vehicle.

To approach the clinical situation, plaque tissue was isolated from large atherosclerotic vessels obtained by autopsy. Cell-free homogenates were prepared from pools of four plaques [5], and these were used as thrombogenic surface for flow chamber studies. Human blood, supplemented with OG488-labeled fibrinogen, was perfused over the plaque homogenate, again under conditions of high shear and coagulation. This resulted in the assembly of large, fluorescent thrombi, which were interconnected by fluorescent fibrin fibers (Fig. 7A). Preincubation of the blood with ticagrelor or cangrelor substantially reduced thrombus size as well as fibrin fiber formation (Fig. 7B, C). That thrombus size was reduced with either P2Y_12_ antagonist was further confirmed by morphometric analysis of the platelet aggregates (Fig. 7D). This was in agreement with analysis of the disaggregation events during blood flow, pointing to a marked destabilization of the thrombi formed in the presence of ticagrelor or cangrelor (Fig. 7E).

**Figure 8:**
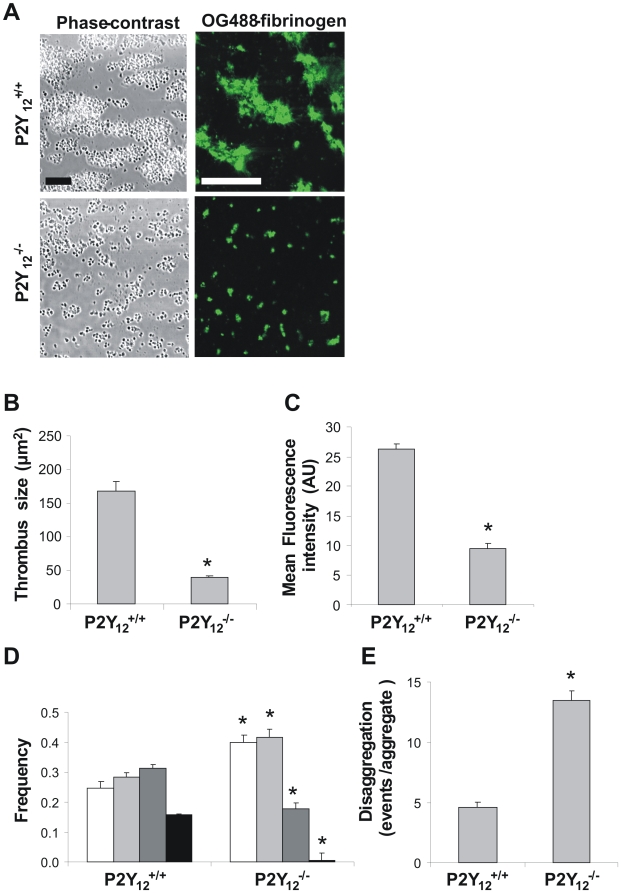
Deficiency of murine P2Y_12_ receptors affects thrombus formation on plaque at high shear rate. Citrate-anticoagulated blood from P2Y_12_^−/−^ and corresponding wild type (P2Y_12_^+/+^) mice was supplemented with OG488-fibrinogen (25 µg/ml) and co-perfused with ADP (10 µM) over homogenized murine plaque material at 1000 s^−1^ for 4 min. (A) Representative phase-contrast and fluorescence images (bar = 20 µm). (B) Average size of thrombi after perfusion. (C) Average fluorescence intensity (arbitrary units) from fibrin(ogen)-binding platelets and thrombi. (D) Histograms of features on surface; estimated numbers of platelets per feature were 9–24 (white), 24–75 (light gray), 75–400 (dark gray) and >400 (black). (E) Disaggregation events measured per platelet aggregate per min. Data are means ± SE (*n* = 3), *p<0.05.

To confirm these results based on pharmacological inhibition of P2Y_12_, flow experiments were performed using blood from P2Y_12_-deficient mice. Blood was perfused over homogenized plaque material, which was isolated from atherosclerotic aortic arches of cholesterol-fed *Apoe* ^−/−^ mice. Perfusion of P2Y_12_-deficient blood over this material resulted in greatly reduced thrombus formation, leaving only a monolayer of platelets at the plaque surface (Fig. 8A). Deficiency in P2Y_12_ also suppressed the deposition of OG488-fibrinogen. Image analysis pointed to a significant reduction in both overall thrombus size (Fig. 8B) and label accumulation (Fig. 8C). Morphometric analysis showed the formation of more smaller aggregates with P2Y_12_-deficient blood (Fig. 8D). These thrombi were instable, as appeared from analysis of the platelet disaggregation events during flow (Fig. 8E).

Altogether these results indicate that ADP, via P2Y_12_, stabilizes thrombi formed under flow on ruptured plaques in the mouse carotid artery *in vivo*, and on human and mouse plaque material *in vitro*. Furthermore, they show that the contribution of P2Y_12_ to thrombus stability increases with shear rate and is maintained in the presence of coagulation.

## Discussion

In the present paper, we used a novel *in vivo* mouse model of thrombus formation on acutely ruptured atherosclerotic plaques, to demonstrate a marked antithrombotic effect of two reversible blocking agents of the platelet P2Y_12_ receptors, *i.e.* ticagrelor and cangrelor. Both P2Y_12_ inhibitors destabilized thrombus formation by increasing the embolization of platelets, with as a result smaller sized thrombi. Our data extend earlier findings, using the same model of acute atherothrombosis, which indicated that thrombus formation is the result of a multi-factorial process involving: platelet interaction with collagen, exposure of tissue factor, generation of thrombin, and formation of fibrin fibers [26]. Recently, other authors have confirmed the importance of collagen and thrombin, using the same model of carotid plaque rupture by ultrasound [30]. Since these *in vivo* data pointed to collagen as a major thrombogenic component in the carotid plaque, we continued to study thrombus formation by *in vitro* perfusion studies with immobilized plaque material or native collagen as a surface. However, it should be noted that also other thrombogenic components have been identified in plaque material, including tissue factor, oxidized low density lipoproteins and lysophosphatidate [8], [10], [31].

A key role of platelet P2Y_12_ receptors in thrombus stabilization was confirmed by *in vitro* flow studies using either mouse or human blood. Markedly, when coagulation was introduced, blockage or deficiency of P2Y_12_ suppressed the formation of larger sized platelet aggregates as well as the formation of fibrin fibers, in particular at high shear rate. Furthermore, inhibition or absence of P2Y_12_ destabilized the thrombi formed on immobilized collagens or plaque material. Also cone-and-plate(let) experiments, where platelets adhered to a surface, showed an enhancing effect of P2Y_12_ on thrombus formation at high but not low shear rate.

In earlier work, we and others have shown that autocrine, platelet-derived ADP contributes to thrombus growth and stability on collagen-containing surfaces [18], [32], [33]. The present data confirm this and indicate that, in addition, the thrombus-stabilizing role of P2Y_12_ is maintained in the presence of coagulation. Mechanistically, P2Y_12_ can contribute to the formation of stable thrombi via two different processes: (i) continuous signaling to phosphoinositide 3-kinases β and γ with as a result sustained α_IIb_β_3_ activation [18]; (ii) enhancement of thrombin/fibrin generation due to increased Gi-mediated Ca^2+^ mobilization and platelet procoagulant activity [20], [21], [34]. The observations that ticagrelor and cangrelor retards ADP-dependent thrombin generation and fibrin formation relates to the latter function. In conjunction with the present results, others have reported that P2Y_12_ is implicated in shear-induced platelet aggregation via activation of Syk kinase and phosphoinositide 3-kinase [35]. Interestingly, another study describes no effect of cangrelor of collagen-dependent thrombus formation [36]. However, this study focused short-term processes (<1.5 min), during which thrombi are still in the growing phase and the contribution of adhesive receptors such as GP-Ib-V-IX is relatively high. Our time-dependent analysis however indicates that a role of P2Y_12_ becomes more prominent at later stages.

The P2Y_12_-directed prodrug clopidogrel is increasingly used to prevent secondary ischemic events in patients with myocardial infarction or stroke [37], [38]. Recently, clinical studies performed with the new, irreversible P2Y_12_ inhibitor prasugrel [39], [40] and the reversible P2Y_12_ inhibitor ticagrelor [41], [42] have shown promising results for the treatment of acute coronary syndrome. Our findings suggest that the success of such anti-P2Y_12_ interventions relies on the selective abrogation of a platelet response–autocrine activation of P2Y_12_–that is at least in part dependent on the local, high shear conditions.

In summary, we have shown that ADP, via P2Y_12_, stabilizes thrombi on ruptured plaques both *in vivo* and on human plaque material *in vitro*. Furthermore, this P2Y_12_ dependency of thrombus stability is maintained in the presence of coagulation, but most pronounced at high shear flow conditions.
